# Fusion of Heterogeneous Intrusion Detection Systems for Network Attack Detection

**DOI:** 10.1155/2015/314601

**Published:** 2015-07-29

**Authors:** Jayakumar Kaliappan, Revathi Thiagarajan, Karpagam Sundararajan

**Affiliations:** ^1^Computer Science and Engineering, Kamaraj College of Engineering and Technology, Tamilnadu 626 001, India; ^2^Information Technology, Mepco Schlenk Engineering College, Tamilnadu 626 005, India

## Abstract

An intrusion detection system (IDS) helps to identify different types of attacks in general, and the detection rate will be higher for some specific category of attacks. This paper is designed on the idea that each IDS is efficient in detecting a specific type of attack. In proposed Multiple IDS Unit (MIU), there are five IDS units, and each IDS follows a unique algorithm to detect attacks. The feature selection is done with the help of genetic algorithm. The selected features of the input traffic are passed on to the MIU for processing. The decision from each IDS is termed as local decision. The fusion unit inside the MIU processes all the local decisions with the help of majority voting rule and makes the final decision. The proposed system shows a very good improvement in detection rate and reduces the false alarm rate.

## 1. Introduction

*Intrusion detection system (IDS)* monitors the behavior of a given environment and identifies the activities that are malicious or legitimate. There are two common approaches to intrusion detection: misuse detection and anomaly detection. Misuse detection via signature verification compares a user's actions with the known signatures of attackers attempting to enter a system. It is useful for finding known intrusion types, but it cannot detect new attacks [1]. Anomaly detection identifies behavior that differs from well-known statistical patterns for users, systems, or networks. Machine learning techniques are used to capture the normal usage patterns and classify the new behavior as either normal or anomalous. In spite of their capability in detecting unknown attacks, anomaly detection systems result in high false alarm rate [2]. Anomaly detection can be combined with signature verification to identify attacks.

*Feature selection* is the most crucial step in constructing any intrusion detection system [3]. A set of attributes or features that are identified to be the most effective are extracted in order to construct a suitable IDS. Identifying the features that are relevant to the learning algorithm is a challenge. In some cases, redundant features can lead to noisy data that distract the learning algorithm and degrade the accuracy of the IDS, and this slows down the training and testing processes. Feature selection is proved to have a high impact on the performance of the classifiers. Experiments show that feature selection can reduce the building and testing time of a classifier.

*Multiclassifier Systems (MCSs)* focus on the grouping of classifiers with heterogeneous or homogeneous modeling backgrounds to give the final outcome. MCSs perform well when there is very sparse data sample for learning. In the scarcity case, MCSs can use bootstrapping methods such as bagging or boosting [4]. MCSs allow training classifiers on a data set's partitions and combining their results using appropriate combination rules. Two canonical topologies work in the designing of MCSs. They are parallel and serial topologies. In parallel topology, each classifier supplies the same input data so that the last decision of the combined classifier result is made on the basis of the outputs of each classifier obtained separately. Alternatively, in the serial (or conditional) topology, each classifier is applied in a certain order implying some kind of grade or ordering over them.

The rest of the paper is organized as follows.  Section 2 enumerates related works. The proposed methodologies are elaborately dealt with in Section 3 with the algorithms for training and testing multiple IDS.  Section 4 discusses the performance evaluation of the experiments in detail with the results.  Section 5 presents the sum-up of the study.

## 2. Related Works

Thomas and Balakrishnan [5] have optimized the performance of IDS using fusion of multiple IDS. The assignment of weight for each IDS is outlined in this paper, and the weights are aggregated to take a correct decision. DARPA 1999 data set is used to evaluate the IDSs which are outdated. It contains more redundant records, and so it affects classifier accuracy. In their method, binary values are used to decide attack or normal. Giacinto et al. [6] proposed a pattern-recognition approach based on the fusion of multiple classifiers for network intrusion detection. It provides a better tradeoff between generalization abilities and false alarm generation. Unfortunately, the performances of fusion rules on unknown attacks show no improvement over the results of the individual networks that are obtained. No fusion rule provides improvements on the performances of the neural network trained on the overall feature set that attains the same performance of oracle. Siraj et al. [7] proposed the Decision Engine of an Intelligent Intrusion Detection System (IIDS) that fuses information from different intrusion detection sensors using an artificial intelligence technique. Like neural networks it cannot do self-learning and self-training. There is no functionality for customizing the standard attack. Parikh and Chen [8] proposed ensemble of classifiers to combine data from various sources and reduce the cost of false alarm. DLEARNIN and DCMS algorithms are used for the abovementioned purpose. In their paper, sum and product rules are not used. Outputs are not directly compared. Giacinto et al. [9] proposed an unsupervised anomaly-based IDS. Combination of one-class classifiers is used in their work for designing each module with distinct features for training. For high values of false alarm rate, the system gives low detection rate. Li et al. [10] constructed a compact data set by clustering redundant data into a compact one. Features are reduced from 41 to 19 using clustering, and the use of ant colony optimization improved the efficiency of intrusion detection. The combination of the critical features used in this method could not distinguish the attackers and normal users. Sung and Mukkamala [11] have removed one feature at a time to carry out an experiment on SVM and neural network. KDDCup'99 data set has been used to verify this technique. For five-class classification, out of 41 features only 19 of the most significant features are used. Li et al. [12] proposed a wrapper-based feature selection algorithm to construct lightweight IDS. They applied a modified Random Mutation Hill Climbing (RMHC) for search strategy and modified the linear SVM for valuation criterion. This method speeds up the process of selecting features and gives a high detection rate for IDS. Since the types of intruders are wider in nature in today's information era, the scope for the designing of improved IDS is high motivating the proposed work.

## 3. The Proposed System

### 3.1. Motivation

With the advent of online business and the social network, the genuineness of the information available in the internet has become a question. Many human and robot based intruders are playing in an aggressive manner to gain advantages of the information. Also the kind of attacks in the Internet is nondeterministic in nature making it very complex task to detect and react. Most of the present day stand-alone intrusion detection systems are not capable of achieving a reasonably high detection rate and low false alarm rate. Most of the existing works on IDSs show distinct performance in detecting a certain class of attack with improved accuracy while performing moderately for the other classes of attacks. It has become possible to obtain a more reliable and accurate decision for a wider class of attacks by combining the decisions of multiple intrusion detection systems.

Nowadays, the processors are working in an unimaginable speed. So combining multiple IDSs is not a big issue in the computation point of view and best-of-breed solutions have been achieved earlier. A better analysis of existing data gathered by various individual IDSs can detect many attacks that currently go undetected. From the literature survey, it is learnt that the usage of appropriate feature selection techniques simplifies the models to make them easier to interpret, shorter the training times, and enhance the generalization by reducing overfitting. The challenges in designing and deploying IDS are increasing due to the wider reach of the Internet services and nonavailability of standard procedure for characterizing the intruders.

### 3.2. The Proposed System Architecture

The anomaly-based IDSs identify the abnormal, unusual behaviors on a network and tag them as attacks. It does not need any specific knowledge. The disadvantage of this method is that it produces more number of false alarms. The signature-based IDS is well versed in detecting attacks that match a predefined pattern, and it produces very minimum number of false alarms and the fusion of signature-based and anomaly-based techniques is done for three main reasons. First, the false alarm rate should be minimum, and it is only possible in signature-based IDS. Second, any IDS has to identify new attacks and it is possible through anomaly-based techniques. Third the idea is that every IDS is efficient in detecting specific types of attack. For example, anomaly-based IDS is suitable for detecting DOS and R2L type attacks, and signature-based IDS is good for detecting U2R and PROBE which can be inferred from Table 6. The fusion of signature-based and anomaly-based techniques will be able to detect more attacks with less false alarm rate. The proposed system consists of a Multiple IDS Unit (MIU) which contains five IDS units following five different algorithms.

**Figure 1:**
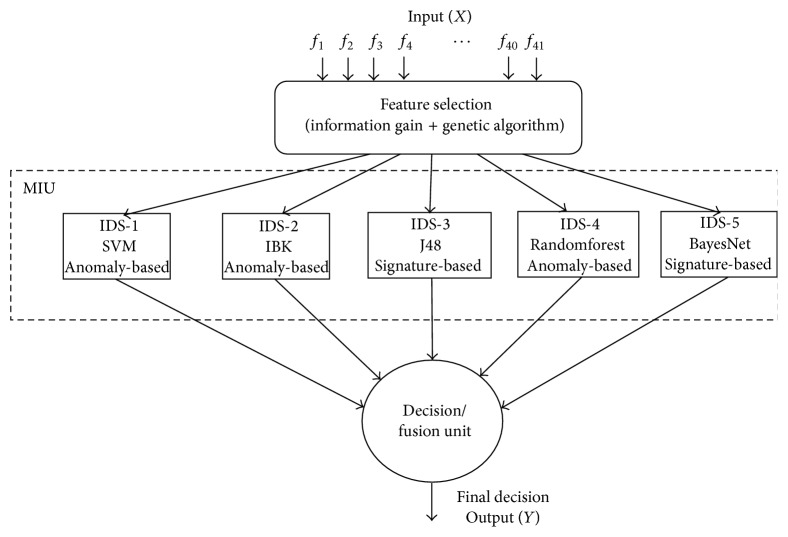
The proposed system architecture.

The proposed system architecture is shown in Figure 1. It contains three phases of work. In the first phase, feature selection is done with the help of information gain (IG) and genetic algorithm (GA). There are totally 41 features present in KDDCup'99 data set. Certain features are irrelevant or not needed for the IDS.

When all the 41 features of the input traffic are taken for processing, there is a delay in processing and inefficient output is produced. Experimenting with all the combinations of the features is exponentially complex in nature. Hence, only the relevant features are chosen with the help of genetic algorithm (Algorithms 1 and 2). The selected features are given as input. The feature selection phase will help in drawing out the relevant features. This increases classifier accuracy and reduces computation speed.

In the second phase, the output from the first phase (i.e., input traffic with selected feature alone) is given as an input to the MIU, and the output is the local decision (*y*_*i*_) which categorizes the input traffic (DOS, PROBE, U2R, R2L, and NORMAL). Five IDSs, each with a unique algorithm, are present in the MIU. The five different types of IDS algorithms used are Support Vector Machines (SVM) [13], IBK, RandomForest, J48, and BayesNet. SVM, IBK, and RandomForest come under the category of anomaly-based IDS [1, 2]. J48 and BayesNet come under the category of signature-based IDS [1]. Every IDS algorithm in the MIU (Algorithm 3) receives the input traffic data record and does the classification for every input record, and five outputs (local decisions) *y*_1_, *y*_2_ to *y*_5_ are obtained.

**Figure 2:**
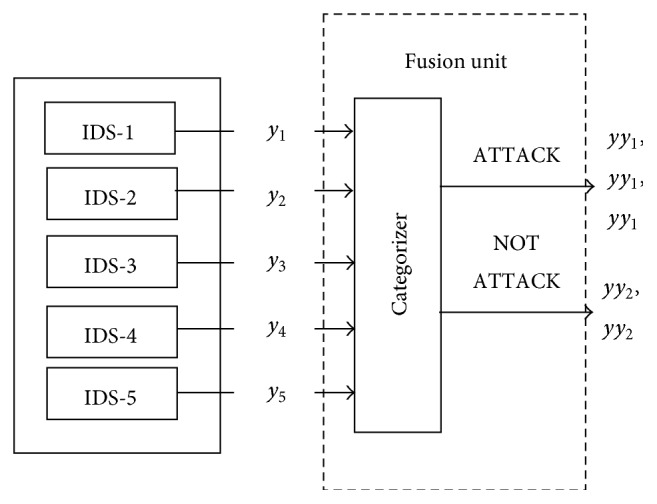
Fusion process.

In the third phase, the output from each IDS_*i*_ in MIU, considered as local decision (*y*_*i*_), is passed on to the categorization unit. The input traffic category is divided into two groups, ATTACK and NOT_A_ATTACK groups. The traffic categories DOS, PROBE, U2R, and R2L are labeled as ATTACK group. Normal is labeled as NOT_A_ATTACK group. For example, if the output (*y*_2_) from the IDS 2 is PROBE, then it falls under the attack group. Fusion process is depicted in Figure 2. The output from the categorization unit *yy*_*i*_ for each local decision (*y*_*i*_) is taken to the decision unit, and the global decision (*z*) is taken based on the majority voting rule. If 3 out of 5 outputs from categorization unit suggest *yy*_1_ (Attack), then the decision unit decides that the input traffic is of ATTACK type; else it is NOT_A_ATTACK.

### 3.3. Feature Selection

#### 3.3.1. Information Gain Ratio (IGR)

Let *S* be a set of training set samples with their corresponding labels. Suppose there are *m* classes and the training set contains *S*_*i*_ samples of class *i* and *S* is the total number of samples in the training set; expected information gain ratio is needed to classify a given sample. It is calculated by using the equation

(1) $$\begin{array}{c} I\left(\;S_{1},S_{2},\ldots ,S_{m}\;\right)=-\overset{m}{\underset{i-1}{\sum }}\left(\;\frac{S_{i}}{S}\;\right)\log_{2}\left(\;\frac{S_{i}}{S}\;\right). \end{array}$$

Feature *F* with values {*f*_1_, *f*_2_, …, *f*_*v*_} can divide the training set into *v* subsets {*S*_1_, *S*_2_, …, *S*_*v*_}, where *S*_*j*_ is the subset which has the value *f*_*j*_ for feature *F*. Furthermore, let *S*_*j*_ contain *S*_*ij*_ samples of class *i*. Entropy of the feature *F* is

(2) $$\begin{array}{c} E\left(\;F\;\right)=\overset{v}{\underset{j-1}{\sum }}\frac{S_{1j}+\cdots +S_{mj}}{S}*I\left(\;S_{1j},\ldots ,S_{mj}\;\right). \end{array}$$

Information gain for *F* can be calculated as

(3) $$\begin{array}{c} \text{IGR}=\text{Gain}\left(\;F\;\right)=I\left(\;S_{1},\ldots ,S_{m}\;\right)-E\left(\;F\;\right). \end{array}$$

#### 3.3.2. GA-Based Feature Selection

To reduce the dimensionality and to get better accuracy, the relevant features have to be selected. Feature selection is done using genetic algorithm. Genetic algorithm fitness function is designed in such a way that the number of features selected has to be minimum and the sum of their information gain value should be maximum. The genetic algorithm is designed to have a population size of 40. The binary chromosome of length 41 is constructed with each bit representing a feature. This binary chromosome is given as input to the fitness function (Algorithm 2). The information gain value (IG) of the selected features (i.e., bit set as 1) is summed up to get the total information gain value (igsum). The total number of 1's set in the chromosome gives the feature count (fcnt). For example, consider the following chromosome:

 11011100011110101100111001110110011010001

Here bit 5 is set (i.e., value = 1); then it indicates that the 5th feature is selected for processing. In this chromosome, totally 24 bits are set, so the feature count (fcnt) is 24. The total information gain value (igsum) obtained by summing up the information gain (IG) of 24 selected features is 0.37586. The genetic algorithm parameter values are listed in Table 1.

Table 2 gives the various eminent feature combinations obtained for different attack types using genetic algorithm. The features that are mostly repeated in the list are selected for the experiment.

The proposed implementation steps are given in Algorithm 3.

## 4. Performance Evaluation and Results

### 4.1. NSL-KDD Data Set

One of the main drawbacks in the KDDCup'99 data set is repetition of records, which causes the learning algorithms to be partial towards the repeated records. Thus it prevents them from learning irregular records which are usually more harmful to networks in U2R and R2L attacks. In addition, the occurrences of these redundant records in the test set will cause biased result in the performance.

The NSL-KDD benchmark data set [14] has the following benefits over the KDDCup'99 data set:

- It does not include repeated records in the training set, and so the classifiers will not be partial towards more repeated records.
- There is no replica record in the testing sets. Therefore, the performances of the learners are not biased.
- The number of selected records from each group of difficulty level is inversely proportional to the percentage of records in the original KDDCup'99 data set and thus helps an accurate evaluation of different learning techniques. As a result, the classification rates of various machine learning methods vary in a wider range, which makes it more efficient to detect different types of attacks. The sample distributions on the training and testing data sets with the corrected labels of NSL-KDD data set are shown in Table 3.

### 4.2. Performance Evaluation Metrics

The performance of the proposed intrusion detection system is evaluated with the help of confusion matrix. The classification performance of IDS is measured by false alarm rate, detection rate, and accuracy. They can be calculated using the confusion matrix in Table 4. Confusion matrix is a 2 × 2 matrix, where the rows represent actual classes, while the columns have the corresponding values to the predicted classes:

(4) False Alarm Rate=FPTN+FP∗100,Detection Rate=TPTP+FN∗100,Accuracy=TP+TNTP+TN+FP+FN∗100.

In this section, the performance of the proposed intrusion detection system is studied with the help of an experiment. In this experiment, only the relevant features are selected, using the information gain algorithm and genetic algorithm. The selected features and training data set are given as input to the MIU unit, and the performance measures such as accuracy, detection rate, and false alarm rate are considered for evaluation. The results are tabulated and plotted as graphs.

### 4.3. Experiment Results

All experiments were performed on a Windows platform having configuration Intel core 2 Duo CPU 2.49 GHZ, 2 GB RAM. Simulations and the analysis of experimental results are performed with the use of Weka machine learning tool [15] and JAVA.

Selected features are considered for training the fusion IDS in this experiment, and test data with 28.39% of novel (new attack) data is taken.

From Table 5 it is inferred that, for J48 classifier, there is 57% of reduction in testing time, when considering 28 features instead of taking all features.

From Table 6 it is inferred that detection rate and false alarm rate of intrusion detection systems with feature selection using single classifier like SVM, IBK, J48, RandomForest, and BayesNet are inferior to those of the fusion IDS unit. For example, in U2R type of attack, the detection rate achieved by SVM classifier is 86%, IBK classifier is 83%, J48 is 82.5%, and BayesNet is 80.5%. When a fusion IDS unit with multiple heterogeneous IDS is used, a higher detection rate of 99% is achieved.

False alarm rate (FAR) is reduced a lot when a fusion IDS unit with multiple heterogeneous IDS is used. For example, the FAR found for DOS attack type using SVM is 0.7, IBK is 0.3, J48 is 0.1, RandomForest is 0.2, and BayesNet is 0.3. When the fusion IDS is used, the FAR is achieved at 0.0.

Detection rate (DTR) and false alarm rate (FAR) of the proposed system for the different types of attack using selected features of the test data set of KDDCup'99 data set are tabulated in Table 7. On an average, 98.4% of detection rate is achieved. The average false alarm rate achieved is 0.68.

The experimental results of Thomas and Balakrishnan [5] paper are taken for a comparative study. Table 7 gives the detection rate of the proposed system and the Thomas and Balakrishnan [5] work. The detection rate for DOS is 64% in previous [5] work and it is 99% for the proposed system. Similarly for PROBE, U2R, and R2L, there is a high improvement in detection rate while comparing with previous work [5]. Particularly for R2L, there is improvement in the detection rate. Similarly, the false alarm rate for DOS is 36.20 in the work of Thomas and Balakrishnan [5], but in the proposed work, the value is minimized to 1.0 and for PROBE, U2R, and R2L also the false alarm rate value has decreased drastically.

**Figure 3:**
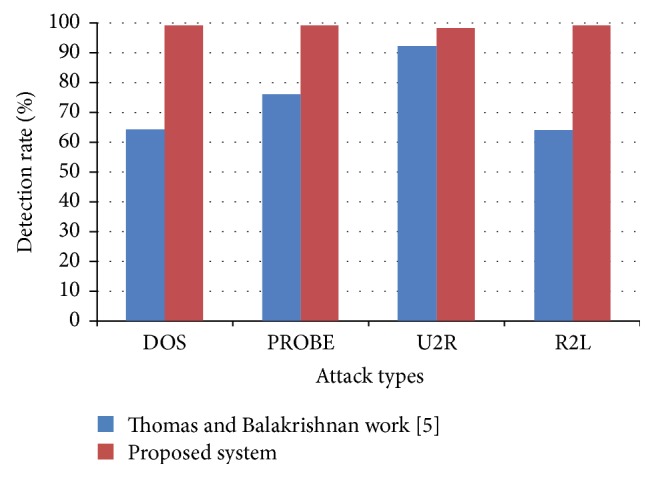
Performance comparison on detection rate of proposed work and Thomas and Balakrishnan [5] work.

**Figure 4:**
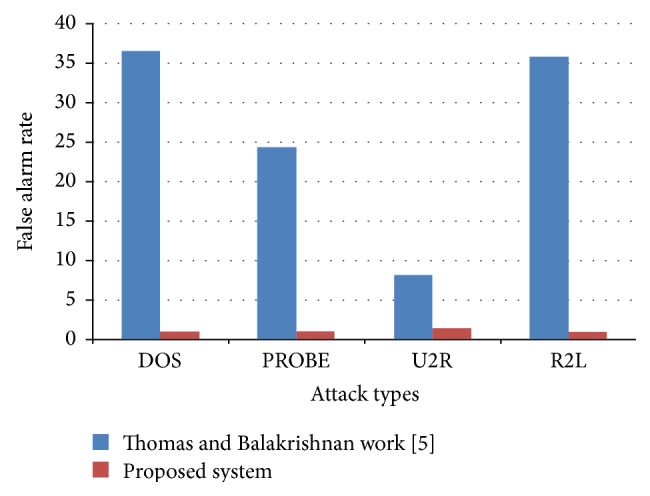
Performance comparison on false alarm rate of proposed work and Thomas and Balakrishnan [5] work.

Figures 3 and 4 present a comparative study of detection rate and false alarm rate of the proposed and existing fusion methods.

## 5. Conclusion

The key idea behind the study is that any IDS is efficient in detecting some specific attack category. Different IDSs which are good in detecting different attacks are combined together, and an MIU is framed. This paper uses only relevant features of the input traffic data for processing, and the promising classification result is obtained from the MIU which is the fusion of heterogeneous IDSs. In comparison with the work of Thomas and Balakrishnan [5], good improvement in the detection rate and false alarm rate is achieved. When the detection rate and false alarm rate of single IDS unit are compared with fusion IDS unit, there is a vast improvement in the performance. The feature selection done with genetic algorithm has extracted the relevant features from the 41 features. As a result, there is improvement in training and testing speed and good accuracy found. The binary interpretation of anomaly score can be avoided in future work. The anomaly score can be normalized and multiplied with the respective weights used as in the basic probability assignments.

## Figures and Tables

**Algorithm 1:**
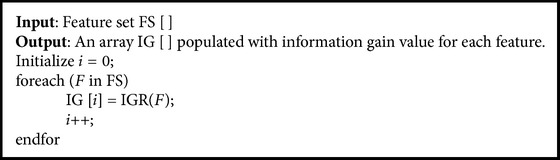
Information gain calculation.

**Algorithm 2:**
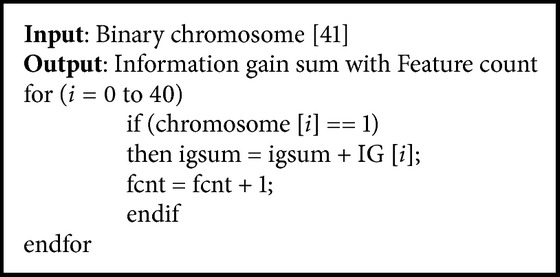
Maximum information gain with minimum feature count algorithm.

**Algorithm 3:**
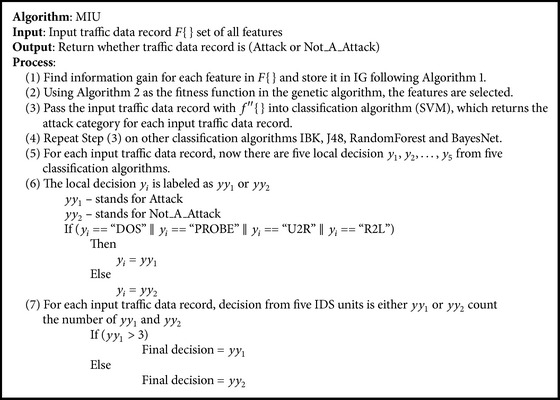
The proposed system algorithm.

**Table 1:** Genetic algorithm parameters.

Modeling description	Setting
Population size	40
Selection technique	Roulette wheel
Crossover type	Uniform crossover
Crossover rate	0.5
Mutation rate	0.1

**Table 2:** Most relevant features for each attack and information gain measures.

Attack type	Attack pattern	Igsum value	Various combination of features giving high information gain value
PROBE	ipsweep	0.82	2, 3, 5, 12, 13, 14, 16, 17, 21, 23, 24, 25, 28, 31, 32, 33, 37, 38
	nmap	0.27	1, 2, 3, 5, 18, 21, 22, 28, 29, 31, 32, 34, 35, 36, 37
	portsweep	0.58	3, 4, 10, 24, 27, 29, 34, 35, 36, 37, 41
	satan	0.75	1, 3, 5, 11, 15, 19, 23, 24, 25, 27, 28, 29, 30, 31, 32, 35, 39, 40, 41
	mscan	1.11	1, 3, 4, 5, 7, 12, 17, 21, 25, 27, 28, 29, 31, 33, 35, 39, 40, 41
	saint	0.33	1, 5, 7, 12, 16, 24, 25, 29, 32, 33, 34, 35, 37, 38, 40

DOS	back	0.38	1, 2, 4, 5, 6, 10, 11, 12, 13, 15, 17, 18, 21, 22, 23, 26, 27, 28, 30, 31, 34, 35, 37, 41
	land	0.0009	1, 2, 3, 4, 7, 13, 18, 25, 29, 35, 38
	neptune	7.73	1, 3, 4, 5, 6, 7, 13, 15, 17, 19, 20, 26, 28, 29, 30, 31, 33, 34, 35, 38, 39
	pod	0.052	2, 3, 5, 7, 8, 9, 10, 11, 17, 19, 21, 23, 26, 33, 34, 39, 40
	smurf	0.68	2, 3, 5, 8, 17, 23, 24, 25, 26, 29, 33, 35, 36, 38, 39
	teardrop	0.27	3, 4, 5, 6, 8, 10, 13, 23, 24, 25, 26, 32, 34, 35, 36, 37, 39, 40

U2R	Buffer_overflow	0.0086	1, 2, 3, 5, 6, 7, 8, 9, 10, 14, 21, 23, 29, 30, 31, 32, 33, 36, 38, 39, 40
	loadmodule	0.0058	1, 2, 3, 4, 7, 8, 14, 27, 36, 39, 40
	rootkit	0.0035	3, 6, 9, 11, 13, 14, 16, 17, 18, 23, 28, 31, 32, 33, 34, 35, 37, 39, 41

R2L	guess_passwd	0.025	2, 3, 4, 6, 9, 10, 11, 13, 14, 17, 21, 23, 24, 37, 38, 39, 40, 41
	imap	0.0035	3, 4, 5, 6, 10, 12, 20, 23, 25, 27, 29, 30, 3233, 34, 36, 38, 39, 41
	multihop	0.0024	3, 4, 10, 12, 13, 14, 16, 17, 18, 19, 22, 26, 27, 30, 35, 37
	phf	0.0021	3, 4, 6, 8, 9, 10, 13, 14, 19, 28, 29, 36
	spy	0.0003	2, 3, 4, 5, 9, 15, 18, 22, 16, 39
	warezclient	0.21	3, 4, 5, 6, 10, 12, 14, 16, 24, 27, 28, 29, 30, 32, 33, 34, 35, 37, 38, 39, 40, 41
	warezmaster	0.008	1, 2, 3, 4, 6, 12, 13, 14, 16, 17, 19, 22, 23, 24, 31, 35, 36, 37, 39

Normal		11.96	1, 2, 3, 4, 5, 6, 7, 15, 23, 24, 14, 15, 19, 20, 21, 23, 25, 26, 27, 28, 30, 32, 33, 34, 36, 37, 38

**Table 3:** The sample distributions on the training and testing data sets with the corrected labels of NSL-KDD data set.

Class	Training data set		Testing data set		
	Number of samples	Samples percentage (%)	Number of samples	Samples percentage (%)	Number of novel attack samples
Normal	13449	53.39	9866	43.76	—
PROBE	2289	9.09	2421	10.74	1315
DOS	9234	36.65	7456	33.07	1715
U2R	11	0.04	67	0.30	32
R2L	208	0.83	2734	12.13	538
	25192	100	22544	100	3600

**Table 4:** Confusion matrix.

	Predicted attack	Predicted normal
Actual attack	True positive (TP)	False negative (FN)
Actual normal	False positive (FP)	True negative (TN)

True positive (TP): the number of attacks detected when it is actually attack.

True negative (TN): the number of normal detected when it is actually normal.

False positive (FP): the number of attacks detected when it is actually normal.

False negative (FN): the number of normal detected when it is actually attack.

**Table 5:** Comparison of training and testing (built-in) time for different classifier using all and selected features.

Classifier	Training data set			Testing data set		
	All features (seconds)	28 features (seconds)	Reduction in training (built-in) time (%)	All features (seconds)	28 features (seconds)	Reduction in testing (built-in) time (%)
BayesNet	0.86	0.47	59	0.69	0.55	23
RandomForest	13.91	10.31	30	12.88	10.07	24
J48	1.92	1.55	22	1.69	0.94	57
IBK	0.30	0.15	67	0.25	0.14	56
SVM	79.0	71.0	11	126.00	121.0	4

**Table 6:** Detection rate and false alarm rate of each classifier for test data.

Attack type	Detection rate					False alarm rate				
	Anomaly-based			Signature-based		Anomaly-based			Signature-based	
	SVM	IBK	RandomForest	J48	BayesNet	SVM	IBK	RandomForest	J48	BayesNet
DOS	95.4	99.5	99.7	99.6	93.7	0.7	0.3	0.2	0.1	0.3
PROBE	98.1	97.7	98.2	98.1	98.0	0.8	0.3	0.1	0.1	1.2
U2R	86.0	83.0	86.0	82.5	80.5	0.1	0.2	0.1	0.1	0.8
R2L	94.3	94.0	95.5	95.2	90.4	1.6	1.1	0.7	0.6	2.3
Normal	94.1	97.2	98.5	98.5	92.1	3.8	1.8	1.2	1.3	2.2

**Table 7:** Comparison of detection rate and false alarm rate for Thomas and Balakrishnan [5] work and proposed system for different attack.

Attack	Detection rate		False alarm rate	
	Thomas and Balakrishnan [5]	Proposed system (28 features)	Thomas and Balakrishnan [5]	Proposed system (28 features)
DOS	64	99	36.50	1
PROBE	76	99	24.32	1
U2R	92	98	8.10	1.38
R2L	64	99	35.84	1
